# Designing Lignin-Based Biomaterials as Carriers of Bioactive Molecules

**DOI:** 10.3390/pharmaceutics15041114

**Published:** 2023-03-31

**Authors:** Turdimuhammad Abdullah, Gülmire İlyasoğlu, Adnan Memić

**Affiliations:** 1 Department of Chemistry, Istanbul Technical University, Istanbul 34467, Turkey; 2 National Research Center on Membrane Technologies, Istanbul Technical University, Istanbul 34467, Turkey; 3 Center of Nanotechnology, King Abdulaziz University, Jeddah 21589, Saudi Arabia

**Keywords:** lignin, drug delivery, biomaterials, hydrogels, cryogels, electrospinning, 3D printing, antioxidant, antimicrobial

## Abstract

There is a need to develop circular and sustainable economies by utilizing sustainable, green, and renewable resources in high-tech industrial fields especially in the pharmaceutical industry. In the last decade, many derivatives of food and agricultural waste have gained considerable attention due to their abundance, renewability, biocompatibility, environmental amiability, and remarkable biological features. Particularly, lignin, which has been used as a low-grade burning fuel in the past, recently attracted a lot of attention for biomedical applications because of its antioxidant, anti-UV, and antimicrobial properties. Moreover, lignin has abundant phenolic, aliphatic hydroxyl groups, and other chemically reactive sites, making it a desirable biomaterial for drug delivery applications. In this review, we provide an overview of designing different forms of lignin-based biomaterials, including hydrogels, cryogels, electrospun scaffolds, and three-dimensional (3D) printed structures and how they have been used for bioactive compound delivery. We highlight various design criteria and parameters that influence the properties of each type of lignin-based biomaterial and corelate them to various drug delivery applications. In addition, we provide a critical analysis, including the advantages and challenges encountered by each biomaterial fabrication strategy. Finally, we highlight the prospects and future directions associated with the application of lignin-based biomaterials in the pharmaceutical field. We expect that this review will cover the most recent and important developments in this field and serve as a steppingstone for the next generation of pharmaceutical research.

## 1. Introduction

Over the past century, there has been major economic, industrial, and population growth [1]. As a result, there are several environmental concerns including increased pollution that have arisen over time from these developments [2]. To offset some of these challenges, at least to some extent, it is becoming increasingly necessary to introduce sustainable, green, and renewable resources in the development of new products [3,4]. For example, increasing the use of some biopolymers (i.e., polymers derived from plant, microorganism, or even animal sources) instead of synthetic materials could lower the energy consumption and associated pollution [2,5]. Furthermore, such approaches could help in developing more circular and sustainable economies [5].

There are many biorefinery processes from which biomass is extracted to produce biopolymers for commercial application [4,6]. However, some of the products and by-products generated from these processes are extremely underutilized. One example that has attracted a lot of attention recently is lignin [6,7]. Only a small fraction (i.e., 1–2%) of the more than 50 million tons of lignin produced annually, as a by-product of the paper industry during cellulose extraction, is then converted into specialty commercial production [8,9]. The remaining lignin (i.e., 98–99%) is simply disregarded as waste or used as a low-cost, low-grade burning fuel that adds little to offset the current pollution [9,10]. However, it is important to mention that lignin is the second most abundant biopolymer from renewable sources in general and the most abundant source of aromatic monomers [11].

The structure of lignin can vary depending on the kind of plant used to source it, its culture conditions, and the manner of lignin extraction [12]. However, it is generally recognized that lignin is composed of a random network of crosslinked methoxylated and hydroxylated phenylpropanoid units (i.e., phenyl propane units of either coumaril, coniferyl, and sinapyl alcohols) [13,14]. The lignin configuration is therefore determined by the proportion of these which have a direct effect on lignin branching and reactivity [15]. Even though there are various functional groups on lignin, due to its high molecular weight and steric hindrances that arise from different configurations, it can be difficult to process for some applications [4,13]. To overcome such limitations, researchers have used chemical modifications to manipulate the lignin structure and reactivity, leading to improved functionality [16]. Ultimately, the physicochemical properties of lignin can directly affect its biological activity [15,16].

**Figure 1:**
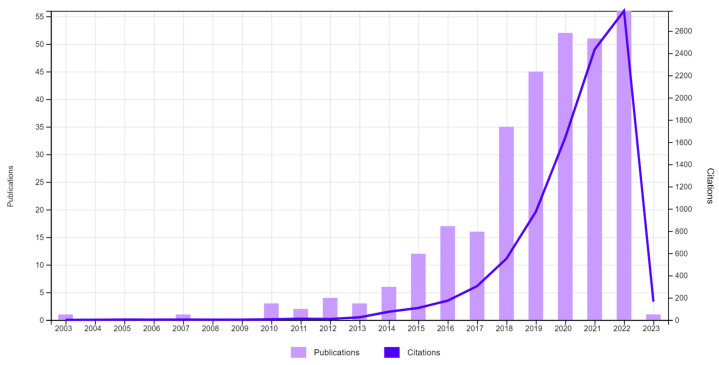
Annual number of publications and citations related to “lignin” and “drug delivery” in the last two decades: data obtained from Web of Science, 13 February 2023.

Lignin has been recognized to have several very advantageous properties, including its antimicrobial and antioxidant nature [15,17]. Similarly, lignin has a capacity for UV and selected chemical adsorption; in addition, it can retain water for long periods, making it an attractive candidate for biomedical applications [15,18,19]. When coupled with biodegradation, low-toxicity, and eco-friendliness, lignin has the potential to be propelled to the forefront of biomaterials research [6,20]. In the past, lignin has been prepared for various bioengineering applications, including biosensing, tissue engineering, and regenerative medicine [21,22,23]. However, recently it has also garnered a lot of interest from pharmaceutical and drug delivery fields (Figure 1). Various lignin-based biomaterials preparations have been proposed that include lignin as the active compound itself or as a carrier of bioactive molecules [6,7,20].

Several review articles have been published in the past to summarize the applications of lignin-based biomaterials in drug delivery, but most of them focus on the nanoparticles-based drug carrier only [24,25,26]. In contrast to nanoparticles, biomaterials in the form of scaffolds can simultaneously serve as supporting materials for defected tissue regeneration and carriers of bioactive molecules. Furthermore, they can deliver large substances, such as cells, genes, proteins, and other supermolecular therapeutic agents. Therefore, in this review, we plan to concentrate on the lignin-based scaffolds and how they have been used for bioactive compound delivery. We plan to cover several biomaterials scaffolds including hydrogels, cryogels, electrospun scaffolds, and three-dimensional (3D) printed structures using lignin and its various derivatives. We plan to discuss various design criteria and parameters that contribute to the development of lignin-based biomaterials with novel and improved properties. We attempt to summarize such developments and provide a critical analysis, including the drawbacks and advantages of using each biomaterial approach. Finally, we provide our perspectives and discuss the prospects and future directions associated with lignin-based biomaterials. Ultimately, we hope that this short review will highlight the most recent and important developments in the field and serve as a steppingstone for the next generation of innovative research.

## 2. Overview of Lignin

As a major component of lignocellulosic biomass, lignin has been considered the second most abundant and annually renewable natural polymer in the world [27]. Approximately 50-e100 billion tons of lignin are renewed in the biosphere every year, yet only 1.5–1.8% is currently produced in industry and is mainly used as fuel to obtain energy [28]. The production cost of lignin is also significantly less than many synthetic polymers such as polyethylene (the most commonly produced synthetic polymer; the price is approximately 1000 USD/dry ton), with prices ranging from 200 to 500 USD per dry ton depending on its purity [27,29]. This makes lignin a promising substitute for many natural and synthetic polymers [29]. There are mainly two types of the extraction process to produce industrial lignin from the lignocellulosic biomass: the sulfur-contained and the sulfur-free processes [30]. Sulfur-contained lignin is mainly extracted from cellulose as a byproduct in the pulp and paper industries, which include Kraft lignin and lignosulfonates [28,30]. The Kraft process involves using a mixture of chemicals such as sodium hydroxide (NaOH) and sodium sulfide (Na_2_S), while the sulfite process involves cooking with an aqueous sulfur dioxide (SO_2_) and a base, such as calcium, sodium, magnesium, or ammonium [30,31,32]. The sulfur-free method is a recently emerged process that utilizes several fractionation steps to create lignin with a high purity and low molecular weight [30]. The chemical composition of these types of lignin is similar to that of native lignin [30,33]. Sulfur-free lignins can be classified into two main categories, namely organosolv lignin obtained from solvent pulping and soda lignin obtained from alkaline pulping [28]. Table 1 provides an overview of the sources, prices, and physicochemical properties of the industrial lignin extracted from different plants.

An assessment of biocompatibility is one of the key concerns for the application of lignin in biomedical fields, such as drug delivery. As a natural polymer derived from plants, lignin is generally considered to have good biocompatiblity and has been shown to have negligible cytotoxicity in many studies [22,34,35]. However, the biocompatibility of different types of lignin can vary because the properties of lignin change when it is extracted through various chemical processes, leading to differences in its physical and chemical characteristics [22,36]. This includes changes in molecular weight, solubility, variations in aromatic content, and the presence of impurities, such as ash and sulfur [22,36]. For instance, the Kraft process, which involves using high temperatures and harsh chemicals, could cause irreversible damage to the highly condensed lignin, resulting in the reduction in ether bond linkages, particularly β-O-4 bonds [37]. As a consequence, the processed lignin from this approach is less chemically reactive and more cytotoxic compared to native lignin and other types of technical lignin [22,37]. Therefore, it is important to conduct studies for each type of lignin to evaluate their biocompatibility [22].

As another important consideration, the biodegradability of lignin has also been studied extensively [38,39,40]. Several studies have demonstrated that lignin can be degraded by a variety of microorganisms, including bacteria and fungi, both in the presence and absence of oxygen [39,41]. These microorganisms can produce enzymes that are capable of breaking down lignin into simpler compounds, which can then be used as a carbon source for growth [41]. The biodegradability of lignin is affected by several factors, including its chemical structure, the source of the lignin, and the conditions under which it is exposed to microorganisms [42]. In general, lignin with a lower degree of condensation is more biodegradable than highly condensed lignin [42]. Various functional groups contained in lignin, including hydroxyl, carboxyl, and methoxyl groups, can also affect its biodegradability depending on their position in the lignin structure [43]. Additionally, lignin from softwoods is generally more intractable to biodegradation than lignin from hardwoods [44]. This is due to the higher degree of condensation and lower concentration of functional groups in softwood lignin. Finally, the conditions under which lignin is exposed to microorganisms, such as temperature, moisture, pH, and the presence of other nutrients, can also affect its biodegradability [42]. Generally, lignin is more biodegradable under aerobic conditions, as oxygen is required for the activity of many lignin-degrading microorganisms [42,45].

## 3. Hydrogel-Based Lignin Carriers

Hydrogels are 3D hydrophilic polymer networks created through chemical reactions of one or more monomers that form linkages between polymer chains, allowing them to absorb water up to hundreds and thousands of times their own dry mass [4,46]. In recent years, bio-based renewable hydrogels have gained a boost in popularity in various biomedical fields, including biomedical implants, tissue engineering, and controlled drug delivery [46,47]. Particularly, in the pharmaceutical field, hydrogels have been extensively used as bioactive drug carriers owing to their supreme biocompatibility, hierarchical structure, ability to provide spatiotemporal control over the drug absorption and release profiles, tunable physicochemical properties, and “smart”-ness [46,48,49,50]. Furthermore, hydrogels can entrap and deliver a wide range of therapeutic agents, such as small molecules, nanoparticles, proteins, enzymes, nucleic acids, and others [46,47].

Lignin is an aromatic polymer with abundant phenolic and aliphatic hydroxyl groups, and it has many chemically reactive sites (such as the C3 and C5 positions of the aromatic ring of lignin), which enable it to form multifunctional hydrogels [4,51]. Typically, all types of lignin have been utilized to fabricate lignin-based hydrogels [51,52,53,54]. In general, lignin has been combined with other polymers via crosslinking copolymerization, crosslinking monomers with grafted lignin, or interpenetrating lignin into a polymer network to produce lignin-based hydrogels. It has also been demonstrated that lignin can be copolymerized with crosslinking agents to directly prepare lignin-based hydrogels [53,54,55]. Recently, Evstigneev [56] also disclosed a patent describing a method to produce a pure lignin hydrogel without adding any copolymer.

The fabrication of lignin-based hydrogels by crosslinking copolymerization can be achieved by both physical and chemical crosslinking [51,57]. The abundant polar sites on the backbone of lignin can be employed for the physical crosslinking of hydrophilic polymers by H-bonding [51,57]. For example, Oveissi et al. [58] crosslinked lignin with hydrophilic polyether-based polyurethane (HPU) to form hydrogels. They confirmed that adding lignin via hydrogen bonding improved the mechanical performance and processability of the hydrogels while maintaining their swelling properties. Zhang et al. [59] used a similar physical crosslinking approach to synthesize a lignin–chitosan–polyvinyl alcohol (PVA) composite hydrogel. Microphotograph investigations revealed that lignin served as a macromolecular crosslinking agent by forming hydrogen bonds with the hydroxyl groups. Lignin has also been combined with other polymer resins through a chemical reaction to create composite hydrogels [51]. For instance, Ciolacu et al. [60] dissolved lignin and cellulose in an alkaline solution and crosslinked them covalently using epichlorohydrin as a crosslinker.

Crosslinking grafted lignin and monomers is another widely used method to prepare lignin-based hydrogel [4,61]. In this method, unsaturated monomers or other functional chemicals were added to the lignin backbone to improve its reactivity [62]. Typically, copolymerization could result from adding a double bond to the lignin structure using an unsaturated monomer [51,62]. Through esterification on the phenolic hydroxyl group of lignin, the unsaturated block was added to the lignin backbone to create an unsaturated grafted lignin. This unsaturated lignin polymer was copolymerized to synthesize hydrogels with additional unsaturated monomers, such as hydroxyethyl acrylate [62]. The resulting hydrogel made of lignin was stated to retain water well.

Additionally, interpenetrating lignin into a polymer network via reversible addition-fragmentation chain transfer polymerization (RAFT) and atom transfer radical polymerization (ATRP) has also been utilized to create lignin-based hydrogels with perfectly aligned structures, designed and controlled properties [11,63]. In this method, creating a lignin-based organic halide as a precursor was the first step [4,63]. Copolymerizing the precursor and monomers was the second step [63]. Graft-from and graft-onto were the two main methods for creating hydrogels in the ATRP and RAFT polymerizations [63,64]. The goal of the “Graft-from strategy” was to create polymers from the active sites found on the polymer’s backbone [65]. The active sites on lignin were used to create the grafted polymers, which were typically used as the backbone polymer [4,65]. In the “graft-onto” procedure, synthetic polymers were combined with lignin by creating covalent links between the terminal groups of the graft polymers and the lignin backbone [4,66].

The characteristics such as pore structure, surface morphology, swellability, biodegradability, and mechanical properties are important parameters to be considered when designing a lignin-based hydrogel for drug delivery and other relevant applications [4,67,68]. It has been proved that the nature, size, and shape of lignin, the lignin concentration, and the fabrication method strongly affect these properties [4]. For instance, the pore size of polyacrylic (PA)-based hydrogels expands with the addition of lignin and possesses a rougher surface morphology compared to pure PA ones. However, if the lignin content exceeds the desired value, the network structure fills with lignin, the pores in the hydrogel are sealed off, and the surface changes from a honeycomb shape to a sheet shape [4,69]. Feng et al. [70] synthesized temperature-sensitive hydrogels comprising acetic acid lignin (AAL) and N-isopropyl acrylamide (NIPAAm) by using N, N’-Methylenebisacrylamide (MBAAm) as the crosslinker and H_2_O_2_ as the initiator during graft polymerization. They examined how the pore size increases with an increase in AAL contents. In another study [71], lignin nanoparticles (LNPs) were dispersed into PVA/chitosan hydrogels instead of bulk lignin. They suggested that the presence of LNPs enables the hydrogel to possess more uniform pores, a larger porosity, and higher pore interconnectivity. Furthermore, the crosslinking density and phenolic contents influence the degradability and microbial resistance of the lignin-based hydrogel. The density and degree of crosslinking reduce the accessibility of ligninolytic fungi and actinomycetes [69,72]. Therefore, hydrogels with high crosslinking strength are more resistant to a microbial attack than hydrogels with low crosslinking strength. Because most ligneous fungi have enzyme systems that directly attack the phenolic substructures in hydrogels, lowering the concentration of the phenolic substructures in hydrogels may help protect them from a fungal attack [69].

**Figure 2:**
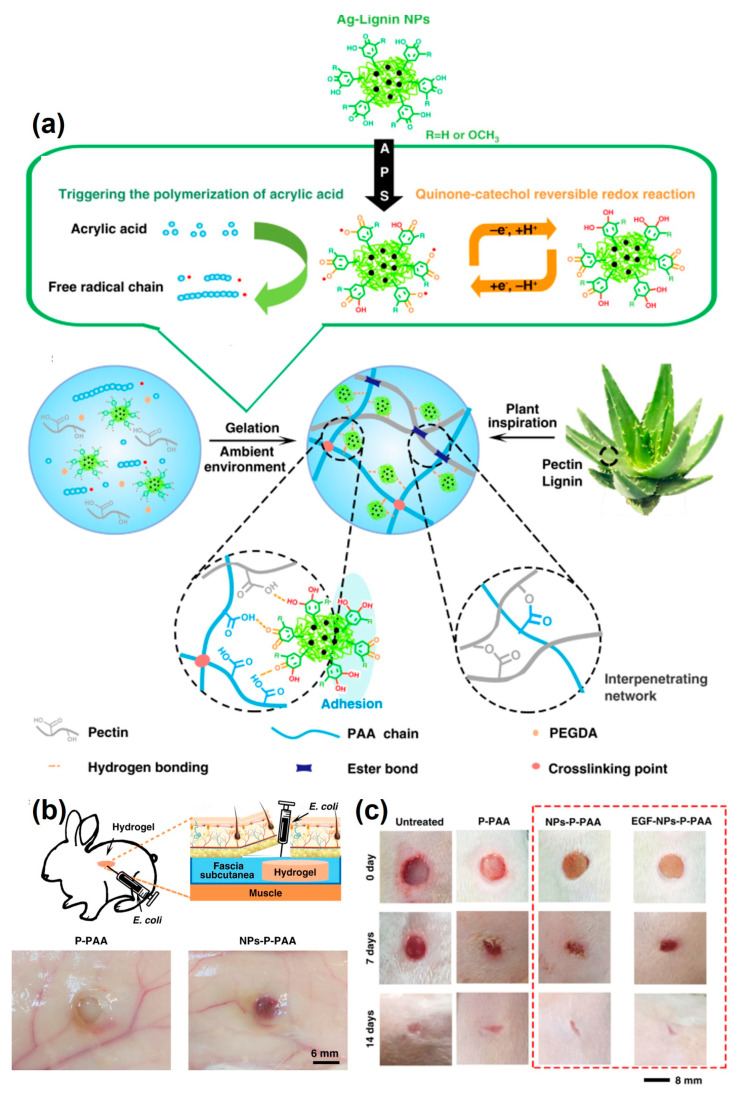
Tough, antibacterial, and adhesive composite hydrogels comprising silver-lignin core-shell nanoparticles (Ag-LNPs), pectin, and acrylic acid (NPs-P-PAA)**.** (**a**) Schematic illustration of the preparation strategy of the composite hydrogels by the lignin-gelatin solution preparation by Ag-LNPs triggered free-radical polymerization. (**b**) Schematic representation of in vivo antibacterial tests and image of hydrogels 7 days after injection of E. coli suspension, in which Ag-LNPs-penetrated hydrogels are free of infection. (**c**) In vivo wound healing test—representative status of wound defects treated with various hydrogels. EGF represents epidermal growth factor (adapted from ref [74] with permission under CC BY 4.0 license).

Lignin-based hydrogels have a broad variety of functional groups to load both hydrophilic and hydrophobic drugs, making them excellent bioactive molecule carriers (Table 2). For instance, Larrañeta et al. [3] applied lignin-based hydrogels to deliver hydrophobic curcumin for cancer treatment. They suggested that the hydrophobic functional groups in lignin enable the hydrogels to load and sustainably release a hydrophobic model drug. In another study [73], lignin-based hydrogels were used as a delivery vehicle of hydrophilic bisoprolol fumarate to alleviate high blood pressure. Gan et al. [74] developed tough, antibacterial, and adhesive hydrogels by penetrating silver-lignin core-shell nanoparticles (Ag-LNPs) into polymeric networks of pectin and acrylic acid (AA). They synthesized Ag-LNPs by a redox reaction, which acted as a triggering agent for the free-radical polymerization of pecten and AA to form composite hydrogels in ambient condition. They further evaluated the application potency of the designed hydrogels in wound healing through in vitro and in vivo experiments (Figure 2).

In numerous other studies, the incorporation of lignin improved the overall drug delivery characteristics of natural polymer-based hydrogels by affecting their morphology, pore structure, and other relevant physiochemical properties. For example, Ciolacu et al. [75] discovered that increasing the lignin content of cellulose-lignin hydrogels (from 25% to 75%) raised the mean hydrogel pore size from 169 m to 431 m and gradually enhanced the drug (i.e., polyphenols) release rate from roughly 17% to 29%. For the regulated release of metronidazole and lysozyme, microcrystalline cellulose was thought to be a high-performance material, and the lignin in the cellulose hydrogel contributed to the controlled release of the drugs [76]. A composite hydrogel that contains both bacterial cellulose (BC)-lignin and the dehydrogenative polymer of coniferyl alcohol (DHP) was developed, and it exhibited an extensive DHP release in the first hour and sustained higher concentrations of antimicrobial chemicals for the following 72 h [77]. Recently, Chiani et al. [55] developed lignin-gelatin hydrogels with different lignin contents to acutely deliver Ribavirin as a COVID-19 treatment. They suggested that the hydrogels with higher lignin content show a higher cumulative drug release due to the improved viscoelastic behavior, pore structure, and swellability.

Regardless of the many desirable traits of lignin-based hydrogels for drug delivery application, several obstacles still need to be addressed. For instance, most natural polymer-based hydrogels, including lignin, suffer from significant drawbacks, such as low mechanical properties and generally quick drug release kinetics. Additionally, the non-adherent nature of hydrogels may need a supplementary dressing to protect them [78]. Furthermore, it is still complicated to construct hydrogels with a pure or high lignin content, due to the polydispersity and uncertain molecular structure of lignin.

**Table 2:** Lignin-based hydrogels and cryogels applied for delivery of various bioactive molecules.

Composition	Fabrication Method	Loaded Drug(s)	Application	Reference
lignin-GAN; lignin-PEG	esterification reaction with microwave radiation	Curcumin	antimicrobial coating	[3]
xanthan-lignin	mixing lignin with xanthan using ECH as crosslinking agent	Bisoprolol fumarate	heart failure/high blood pressure	[73]
cellulose-lignin	mixing cellulose alkaline solution with lignin, followed by the crosslinking with ECH	Polyphenols	cosmetic and pharmaceutical applications	[75]
cellulose-lignin	co-dissolution of polymers in [Emim][Ac], then reconstitution with water.	Lipase	biocatalysts	[79]
PVA/LA	amino group was grafted onto sodium lignin sulfonate (LA), then crosslinked with PVA	Silver nanoparticles	wound healing	[80]
NPs-P-PAA	Ag-LNPs were synthesized by a redox reaction, which acted as a triggering agent for free-radical polymerization of pecten and AA to form composite hydrogels	Silver nanoparticles	wound healing	[74]
BC-lignin	mixing lignin with BC	DHP	chronic wound healing	[77]
alkaline and organosolv lignin	physically crosslinking and chemical modifications	Quercetin	controlled drug delivery	[15]
lignin-gelatin	mixing gelatin alkaline solution with lignin, followed by the crosslinking with EDC	Ribavirin	COVID-19 treatment	[55]
lignin-PVA (cryogels)	blending lignin with PVA via two routes of cross linking	Methylene blue	biocatalysts	[81]
lignin-co-Gelatin (cryogels)	coherently mixing lignin with gelatin and chemically crosslinking	Ag2O/CuO nanoparticles	wound healing and tissue engineering	[82]
LNP@ nanocellulose (cryogels)	anchoring lignin nanoparticles (LNPs) to the nanocellulose network via electrostatic attraction	Diclofenac, metoprolol, tramadol, carbamazepine	adsorbent in environmental engineering	[83]

## 4. Cryogel-Based Lignin Carriers

In addition to traditional hydrogels, there is a subclass of scaffolds that has attracted a lot of attention, made by controlled crosslinking at subzero temperatures and known as cryogelation [84,85,86]. Although cryogelation approaches have been around since the 1940s, it is only in the last couple of decades that they have been popularized for biomedical applications [85,86]. These types of hydrogels are often referred to as cryogels due to the unique properties they possess as a result of their fabrication process [86,87]. One of the major advantages of cryogels over their hydrogel counterparts is that they have a highly interconnected pore network structure coupled with macro-sized pores [88,89]. As a result, cryogels can have highly crosslinked, dense polymer walls that can not only act as drug storage depots but also improve the scaffold mechanical properties and support syringe injectability and shape memory [87,88].

As mentioned in the earlier section, to generate lignin-based macroporous hydrogels, researchers have used approaches such as salt leaching and gas foaming; however, these methods can yield toxic by-products and have a costly multi-step synthesis [90,91]. Overcoming these limitations is possible by utilizing lignin-based cryogels. There are two distinct cryogelation approaches: (i) scaffolds made by multiple freeze-drying cycles using traditional lignin hydrogel precursors (i.e., freeze-drying approach) whereby each freezing cycle is meant to generate additional hydrogen/electrostatic bonds and strengthen the polymer network and (ii) scaffolds made in a frozen solvent via a single-step cryopolymerization step (i.e., freeze-thawing approach) that crosslinks the polymer network [86,92,93]. In both cases, the frozen solvent (i.e., aqueous solutions in many cases) acts as the porogen that leaves behind large pores upon the ice thawing [86,94]. There are several important parameters to consider during cryogelation. For example, varying the temperature of the cryogelation and polymer concentration, the source of the lignin, and its modification can all effect the scaffold properties, including the pore size, shape, and interconnectivity as well as the drug adsorption capacity [94,95,96].

**Figure 3:**
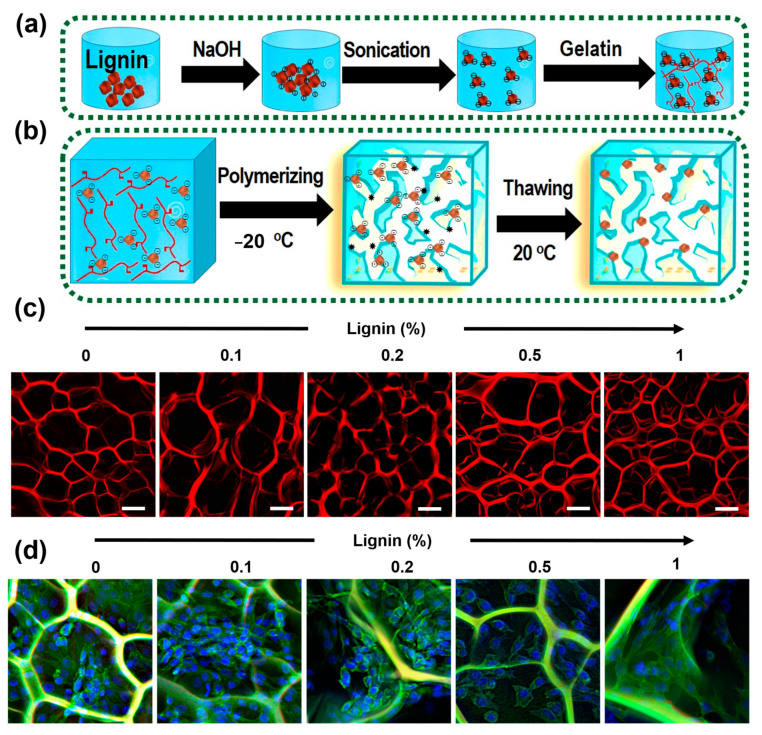
Syringe-injectable antioxidant and antimicrobial lignin-co-gelatin cryogels for biomedical applications. (**a**) Schematic illustration of the lignin-gelatin solution preparation by alkali-assisted ultrasonication method. (**b**) Schematic representation of lignin-co-gelatin cryogel fabrication by freeze-thawing method. (**c**) Zone inhibition diameters of the composite cryogels with different concentration of lignin against *E. coli* and *S. aureus.* (**c**,**d**) Confocal microscopy images of the composite cryogels with different concentration of lignin before (**c**) and 72 h after (**d**) culturing NIH/3T3 fibroblast cells (adapted from ref [96] with permission).

One recent study led by Morales et al. [97] developed a factorial design model for cryogel scaffold fabrication. They varied the concentrations of lignin and PVA polymers using cyclical freeze-drying-based crosslinking and curing methods. The goal of their model was to correlate and improve the swelling rate/capacity of scaffolds and produce minimal lignin waste. Their optimized fabrication protocol enabled fabricating physically crosslinked freeze-dried hydrogels (i.e., cryogels) with an up to 800% water retention capacity that could have implications in controlled drug loading and delivery. Similarly, the same group also studied the effect of the PVA molecular weight and freeze-drying cycle time on scaffold properties (i.e., swelling and lignin waste) [81]. They showed that the higher molecular weight of the blending polymer (i.e., PVA) generated larger scaffold pores while only slightly affecting the thermal properties while requiring fewer freeze-drying cycles. Although they could also improve the mechanical properties of the scaffolds (i.e., compression modulus) by increasing the PVA molecular weight, these properties were not retained upon the addition of lignin. However, the scaffolds did present an improved methylene blue adsorption capacity that could also be translated to other positively charged bioactive compounds. In general, the scaffolds exhibited a pH and temperature responsiveness that could potentially be used for generating “smart” biomaterial scaffolds. The same researchers showed that the lignin extraction method affects scaffolds’ properties (i.e., swelling capacity and compression strength) [98]. Oddly, they observed that lower purity lignin precursors yield scaffolds with highly porous architectures. Furthermore, when tested against the *Aspergillus niger* strain, lignin cryogels showed good antifungal activity (i.e., up to 58% of FGI). In a similar setup, the researchers also showed that lignin-based cryogels have a promising drug loading and release capability [15]. However, other groups have focused on other types of cryogelation. For example, Grishechko et al. [99] developed cryogels and aerogels based on lignin and phenol crosslinking using formaldehyde. They showed that both types of scaffolds had similar properties (i.e., pore size and pore volume) and could be incorporated with up to 80% lignin and had potential applications as thermal insulators. Abdullah et al. [96] fabricated multifunctional, bioactive lignin-co-gelatin composite cryogels by chemically crosslinking lignin together with gelatin at −20 °C (Figure 3). The addition of lignin enabled the cryogels to possess antimicrobial and antioxidant properties, as well as remarkably improved their mechanical properties, syringe injectability, and in vitro biocompatibility. They suggested that the designed platform could be an excellent candidate for various biomedical applications, including wound healing and tissue engineering. They further received a US patent on this interesting work [82].

Despite the development of novel lignin-based cryogels, there are still challenges that need to be addressed. For example, lignin cryogels made via cyclic freeze drying usually possess lower structural stability and significantly weaker mechanical properties [99,100]. As mentioned above, some of those limitations can be addressed using cryopolymerization to fabricate lignin gels [96]. However, although the mechanical properties are significantly improved (i.e., able to be syringe injected and have shape memory and sponge-like behavior), other challenges such as controlled biodegradation still remain which have important implications for drug delivery [101]. Overall, scaffold fabrication via cryogelation can also be advantageous because it requires lower polymer initiator concentrations than their hydrogel counterparts, leading to a lower cost for scaffold production [102]. Alternative fabrication methods such as cryopolymerized physically (i.e., not chemically) crosslinked gels could be useful as well as that would combine some of the advantages of previous approaches, including a reversible gel formation [101,103]. However, close attention must be given to the rate of crosslinking, ensuring that physical crosslinks only take place after freezing and ice formation/growth (i.e., slow processes) [101].

## 5. Electrospun-Based Lignin Biomaterial Carriers

**Figure 4:**
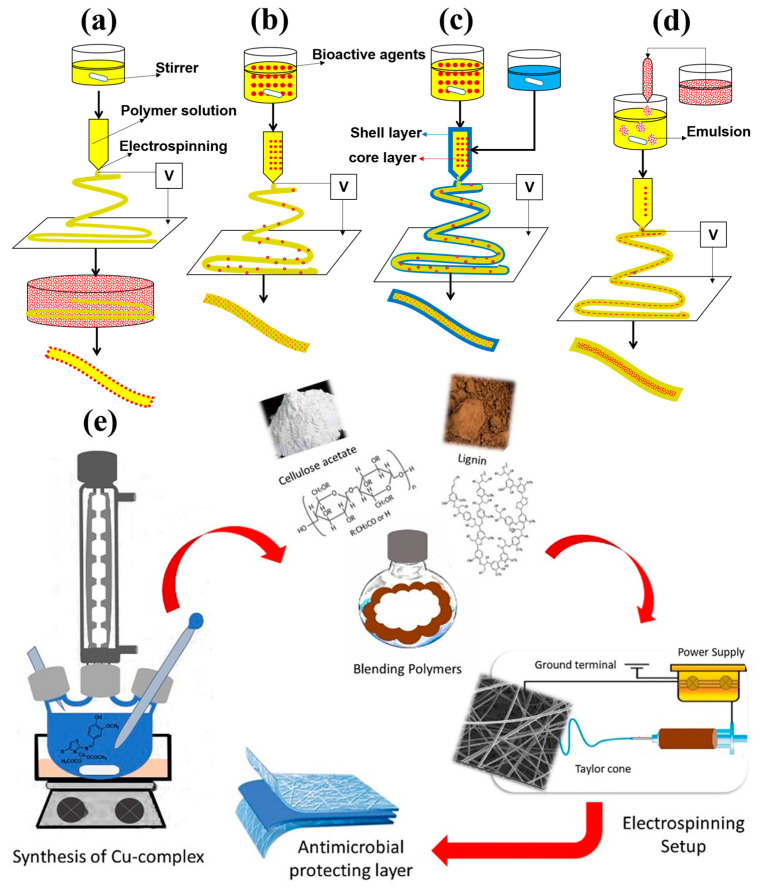
(**a**–**d**) Schematic illustration of different strategies to encapsulate bioactive molecules within the scaffolds fabricated by electrospinning, (**a**) physical or chemical conjunction, (**b**) initial blending, (**c**) coaxial electrospinning, and (**d**) emulsion electrospinning (adapted from ref [109] with permission). (**e**) Schematic illustration of designing bioactive tri-component nanofibrous mat by electrospinning for diaper dermatitis control (adapted from ref [131] with permission).

In recent years, electrospinning has also been extensively investigated for developing a new generation of drug delivery systems [104,105,106]. It is a nifty and flexible technique to create a nanofibrous network that closely resembles the native extracellular matrix (ECM) [107,108]. The basic electrospinning setup comprises a high-voltage electrical supply, a syringe pump, and a collector [109]. Typically, the polymer solution or melts delivered to the spinneret is polarized by an electric charge in the form of a Tailor cone and eventually ejects and loses its solvent to generate ultrathin fibrous mesh on the collector [109]. The formation, dimension, and surface morphology of the nanofibers are regulated by several electrospinning parameters, such as the applied voltage, viscosity, feed rate, and fiber collection method [109,110]. Many desirable traits of electrospun scaffolds, such as a high surface area, high porosity, pore interconnectivity, resistance to agglomeration, and high drug loading and/or encapsulation capacity, make them excellent candidates for drug delivery and other biomedical applications [104,111]. In addition, electrospinning offers numerous strategies to immobilize the bioactive molecules, including but not limited to chemical or physical conjugation, initial blending, emulsion electrospinning, and coaxial electrospinning (Figure 4a–d) [112]. In this way, it is possible to incorporate a wide variety of therapeutic agents within the fibrous mesh regardless of their miscibility [109,113]. Furthermore, the drug release profile and electrospun scaffold properties can be easily tuned by adjusting the electrospinning parameters or applying different drug encapsulation methods [109,114].

Despite these advantages, it is challenging to electrospin pure lignin due to its heterogenic chemical composition, complex branched structure, and low molecular weight [115,116]. Therefore, in most cases, lignin has been combined with other hydrophilic or hydrophobic polymers to generate the lignin-based electrospun nanofibrous structure [116]. Polylactic acid (PLA) [117], polyvinylpyrrolidone (PVP) [118], polyvinyl acetate (PVA) [119,120], polycaprolactone (PCL) [121,122], PEO [123,124], and polyhydroxybutyrate (PHB) [125] are some of the most common polymers used as a carrier and/or dopant to improve the spinnability of lignin. Nevertheless, a few studies reported the possibility of electrospinning pure lignin without adding any carrier polymers [126,127,128]. Lallave et al. [126] fabricated pure lignin nanofibers by a coaxial electrospinning technique, in which the inner layer solution of Alcell lignin in ethanol was sheathed by ethanol to prevent the rapid evaporation of the solvent. Recently, Parot et al. [127] successfully generated bead-free, uniform organosolv lignin nanofibers in a basic electrospinning device by dissolving the lignin in *N*,*N*-dimethyl formamide (DMF) and simply optimizing the electrospinning parameters.

In the past, the majority of studies were devoted to using lignin-based electrospun nanofibers for producing carbon nanofibers for electrochemical storage, sensors, or other related applications [116,129]. However, recent studies showed that the electrospinning of lignin-based materials could also be used for drug delivery and other advanced biomedical applications (Table 3) [23,130,131,132]. Morganti et al. [23] applied electrospinning to produce nanofibrous beauty masks containing chitin-lignin nanoparticles that can entrap and sustainably release many bioactive compounds, such as melatonin, vitamin C, and beta-glucan. They further disclosed an invention describing this process [123]. Aadil et al. [130] synthesized lignin-stabilized silver nanoparticles (AgNPs) and added them into a blend solution of PVA/lignin to fabricate antimicrobial nanofibers by electrospinning. They revealed that active functional groups present in lignin, such as phenolic, methoxyl, and carboxyl groups, could effectively reduce silver ions to metallic silver during the synthesis of AgNPs. Abdullah et al. [132] demonstrated the potency of PCL-coated chitin-lignin nanofibrous scaffolds in drug delivery-based wound dressing applications. They encapsulated the chitin-lignin core layer with a hydrophobic PCL shell layer by a coaxial electrospinning technique to prevent the immediate release of the drugs within the chitin-lignin electrospun scaffolds. They showed that the antibiotics-loaded core-shell fibrous scaffold could effectively retard the invasion of numerous pathogens responsible for hospital-acquired infections, such as *S. aureus* and *E. Coli.,* without causing any significant cytotoxicity. They further illustrated a series of scaffolds that can simultaneously release drugs and oxygen in their granted patent [8]. Recently, Elsherbiny et al. [131] blended several copper (Cu) complexes with a lignin/cellulose acetate (CA) solution and electrospun them to generate antimicrobial nanofibrous mats for hygienic applications (Figure 4e). All the Cu complexes were effectively encapsulated within the lignin/CA nanofibers and showed strong and durable antimicrobial activity against several skin pathogens, including *Staphylococcus epidermidis*, *Acinetobacter baumannii*, *Pseudomonas aeruginosa*, and *Streptococcus faecalis*. Furthermore, the good compatibility of nanofibrous mats with dermal tissues and their improved moisture absorption capacity, which is proportional to the lignin content, could make them a promising candidate for diaper dermatitis control.

In several reports, lignin itself served as a therapeutic agent, owing to its inherent antimicrobial, antioxidant, and anti-inflammatory properties [121,124,135]. For example, Wang et al. [121] showed that the addition of lignin within PCL nanofibrous scaffolds could endow them with excellent antioxidant properties; enhance the viability, propagation, and the differentiation of Schwann cells; and ultimately facilitate nerve tissue generation. Similarly, Liang et al. [135] evaluated the applicability of the antioxidant PLA/lignin nanofibrous scaffold for cartilage tissue engineering and osteoarthritis treatment. They also suggested that the scaffolds can be used in many other biomedical applications, including UV filtration and consumer care. Overall, electrospinning provides tremendous opportunities to design a variety of lignin-based biomaterials with multifunctionality and pertinent physicochemical properties for pharmaceutical and other medical applications [104,106]. Nevertheless, there are certain limitations in transferring this technique to the pharmaceutical industry, including the need for a high-voltage environment, the evaporation of tons of toxic solvents, and a low yielding speed [104,136]. Furthermore, electrospinning generally fabricates 2D dense fibrous scaffolds with limited cell infiltration that poorly simulate living organisms [86,137]. In this regard, coupling electrospinning with other advanced manufacturing techniques (e.g., 3D printing and cryogelation) could be a trending topic to utilize nanofibers more efficiently for drug delivery applications [85,136,137].

## 6. Three-Dimensional Printing-Based Lignin Biomaterial Carriers

Additive manufacturing (AM) or 3D printing is a digital manufacturing process to fabricate end-use products layer by layer with customized sizes, shapes, and functionality using computer-aided design (CAD) [138,139]. Since its invention in 1981, many types of AM techniques, such as stereolithography (SLA), digital light processing (DLP), direct ink writing (DIW), fused deposition modeling (FDM), selective laser sintering, and selective laser melting, have been established to print broad ranges of materials, ranging from polymers to metals and ceramics [140,141]. In addition, many innovative AM techniques, such as 4D/5D/6D printing [142,143,144], melt electrowriting (MEW) [145], and cryoprinting [146], are continuously emerging to advance this technology to new heights. Currently, it has become one of the fastest developing technologies that is expected to ultimately replace many traditional manufacturing industries [147,148]. Particularly, AM could provide an opportunity to reform pharmaceutical industries by offering mass-customized and personalized medicine manufacturing platforms that can be easily adapted to customer needs and the market size [149,150]. By simply modifying the CAD file, AM allows the on-demand production of pharmaceuticals with patient-specific dosages [151,152], customized drug formulations [153,154], the most desirable geometrics [149,150], and controllable drug release profiles [155,156]. Furthermore, the scalability of the printers and advances made in in situ printing allow AM to be readily available on the front line (e.g., in hospitals or medical care centers) for printing more tailored personalized medicine or delivering therapeutic agents to hard-to-reach areas of the human body [149,157,158].

Since the last decade, substantial efforts have been made to exploit AM for lignin-based materials, yet none of the currently available AM techniques are suitable for printing pure lignin [29,159]. Therefore, lignin has been either derivatized or combined with other polymers or monomers as fillers or additives to create printable feedstocks [159]. Among the different categories of AM, material extrusion and vat photopolymerization are the most commonly used technologies for printing lignin-based biomaterials [29]. In extrusion-based printing, such as FDM and DIW, the materials are dispensed through an orifice or nozzle to create a 3D structure [160]. It could probably be the most simple and cost-effective AM technology, yet it suffers from a low resolution, low accuracy, and slow printing speed [161,162,163]. On the other hand, vat photopolymerization, such as SLA and DLP, creates 3D parts by selectively solidifying a liquid resin through a rapid polymerization of photoactive monomers using a specific wavelength of light [164,165]. Compared to extrusion-based printing, it provides a more delicate structure and superior surface quality and can print a small-sized item, even down to a micrometers scale [29,164]. However, until now, it is only applicable to limited types of monomer resins [29].

**Figure 5:**
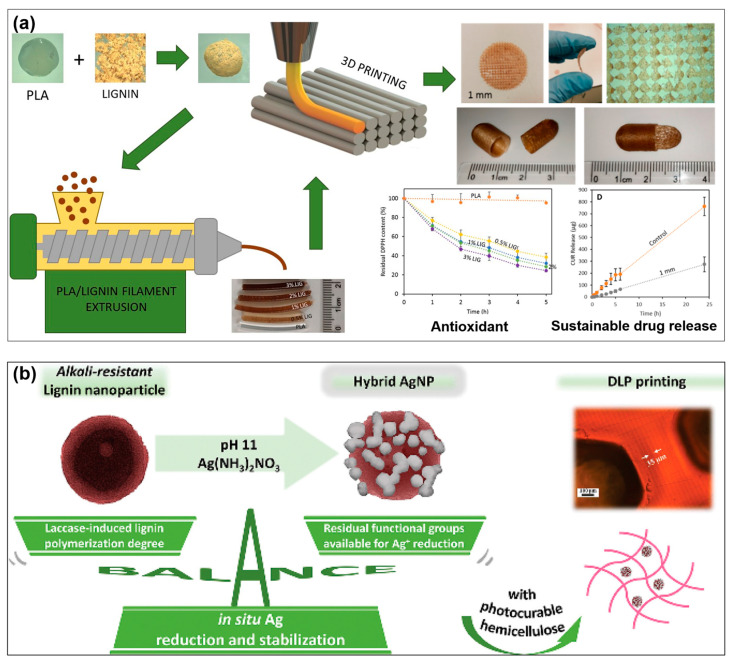
(**a**) Schematic illustration of FDM printing antioxidant PLA composites containing lignin and their applications in sustained drug release (adapted from ref [168] with permission). (**b**) Schematic illustration of DLP printing an antimicrobial hydrogel via integrating lignin nanoparticles (LNPs) decorated by AgNPs (Ag@LNP) in a methacrylated O-acetyl-galactoglucomannan (GGMMA) network (adapted from ref [167] with permission).

In the last few years, lignin-based 3D printed biomaterials have also started to be applied in the pharmaceutical field (Table 4) [166,167,168,169]. Domínguez-Robles et al. [168] produced lignin-coated PLA pellets and printed them by FDM into various mesh sizes containing drugs (Figure 5a). They suggested that the incorporated lignin can effectively reduce the concentration of reactive oxygen species and also slow the release of the model drug. Abdullah et al. [166] developed a series of antimicrobial and antioxidant filaments composed of lignin and polybutylene succinate (PBS) for FDM-based printing, and then further coated them with silver/zinc oxide (Ag/ZnO) to create the strain-specific antimicrobial properties. They found that the printed scaffolds with the highest content of lignin and containing both Ag and ZnO exhibit a strong inhibitory effect against a large variety of microorganisms, ranging from bacteria to fungi. The researchers raised the possibility that lignin could serve in a templating role to stabilize Ag/ZnO as nanoparticles (NPs), prevent agglomeration, improve the diffusion of metal ions, and provide synergetic antimicrobial effects. Wang et al. [167] designed an antimicrobial hydrogel using DLP printing via integrating lignin nanoparticles (LNPs) decorated by AgNPs (Ag@LNP) in a methacrylated O-acetyl-galactoglucomannan (GGMMA) network (Figure 5b). They synthesized alkaline-resistant LNPs via laccase-catalyzed lignin polymerization, which served as both reducing and stabilizing agents for the AgNPs to produce a hybrid antimicrobial Ag@LNP nanocomposite. The incorporation of Ag@LNP within the GGMMA not only greatly improved the printability of the photocurable resin but also enabled the printed hydrogel to possess a strong bactericidal performance. Recently, Domínguez-Robles et al. [169] designed a bioactive wound dressing by loading curcumin (CUR) and D-Panthenol (DPA) onto PCL/lignin in 3D printing. In this platform, CUR was used as an anti-inflammatory and antimicrobial agent, while DPA was included in the formulation due to its high epidermal differentiation ability. The printed PCL/lignin composite offered a sustainable CUR and DPA release and displayed antioxidant, antimicrobial, and anti-inflammatory properties. Both the in vitro and in vivo evaluation results confirmed that the designed dressing shows remarkable improvement at all stages of the wound healing process.

In summary, various AM approaches opened a brand-new research direction for developing lignin-based advanced bioactive carriers. However, the successful translation of this approach in clinical applications requires addressing multifarious technical and regulatory challenges [149,170,171]. For instance, the currently available printers mostly apply extreme conditions, such as high temperatures and laser light, to produce 3D printed structures, which could deteriorate the therapeutic efficacy of the loaded drugs [170,172]. In addition, there is a risk of misusing this technology to fabricate unlicensed medicines or violating good manufacturing practice policy [149,171]. Furthermore, because AM is a disruptive technology, it is critical to create a multisectoral research environment for the AM-based pharmaceutical industry by collaborating with a wide range of contributors to this field, including clinicians, pharma, engineers, scientists, and even patients [149,173].

**Table 4:** Lignin-based 3D printed scaffolds applied for delivery of various bioactive molecules.

Composition	Fabrication Method	Loaded Drug(s)	Application	Reference
Lignin/PLA	FDM	tetracycline	Wound dressing	[168]
GGMMA-LNP@Ag	DLP	AgNPs	Antimicrobial materials	[167]
Lignin/PBS	FDM	Ag/ZnO	Antimicrobial materials	[166]
PCL/lignin	FDM	curcumin and D-Panthenol	Wound dressing	[169]
PLA/lignin	FDM	curcumin	Wound healing	[168]
TPU/lignin	FDM	levofloxacin	Soft tissue reinforcement	[174]

## 7. Conclusions and Future Direction

In this review, we summarized how lignin-based biomaterials could be used for various bioactive compound carrier applications. We first introduced lignin as one of the most abundant natural polymers with a chemical structure that is a great source of aromatic moieties, followed by some considerations to make lignin more useful and reactive. We then delved into specific examples and various lignin biomaterial formulations while focusing on how they can be used for bioactive compound delivery. Our first section on lignin-based biomaterial focused on hydrogels built using lignin and its derivatives. We highlight the main approaches to fabricating lignin-based hydrogels (i.e., crosslinking mechanisms) and their effect on hydrogel and drug release properties. Similarly, we next focused on cryogels, which are a specific type of hydrogel made during crosslinking at subzero temperatures. We discussed how cryopolymerization can be used to overcome challenges associated with the fabrication of traditional lignin-based hydrogels. We focused on cryogel properties, including pore size, degradation, injectability, and network interconnectivity, as ways to tune the activity of lignin itself or the bioactive compound release. Next, we discussed electrospun scaffolds using lignin precursors as another strategy to develop biomaterials scaffolds for controlled delivery applications. We highlighted that during electrospinning it is possible to tune the fiber properties of lignin-based scaffolds which can directly correlate with the carrier release properties. Further tuning is possible by fabricating multilayered micro- and nanofibers that could provide an enhanced on-demand release of bioactive compounds. Finally, we discuss the emerging field of (bio)printing using polymeric lignin precursors to build 3D scaffolds and structures. We discussed how lignin filaments can be fabricated while also potentially incorporating bioactive compounds within the filament itself. Alternatively, larger 3D printed structures could be fabricated to serve as capsules for oral drug delivery applications. Taken together, the versatility of lignin-based biomaterials makes them a very attractive precursor for fabricating a variety of bioactive carriers, including for controlled drug release applications. However, that is not to say that many challenges do not remain to be solved to achieve the full potential for lignin valorization.

Although much progress has been made using lignin-based biomaterials for controlled bioactive compound delivery for certain applications, a larger gap remains. For example, in applications related to food and pharmaceutical fields, the toxicity profiles might require more in-depth, long-term analyses due to the phenol nature of lignin biomaterials. This is further compounded by the non-homogeneous, polydisperse nature and varying molecular weight and structure of lignin when used as a biomaterial during scaffold fabrication. This challenge usually stems from the highly biodiverse sources and lack of standardized extraction procedures for lignin biomaterials. Although lignin-based biomaterials can be tuned to display unique and enhanced properties, a definite gap emerges when compared to the amount of physicochemical data available for other biomaterials. Therefore, it could be more challenging to optimize lignin for some specific clinical applications that are more complex. For example, detailed structure/function relationship studies are required to better understand the mechanism and behavior of lignin-drug carrier interactions. Furthermore, lignin biomaterials might require sterilization, in vivo biodegradation, and biocompatibility studies to better assess the role of lignin on cells and tissues. Recently, artificial intelligence approaches have been emerging as a way to quickly screen for suitable biomaterials; however, anticipating lignin behavior is complex given the diverse nature of lignin biomaterial sources.

## Figures and Tables

**Table 1:** Overview of sources, prices, and physicochemical properties of industrial lignin extracted from different plants.

Types of Lignin	Lignosulfonates	Kraft Lignin	Organosolv Lignin	Soda Lignin
Sources [30]	Softwood; hardwood	Softwood; hardwood	Softwood; hardwood; annual plants	Annual plants
Sulfur content (%) [28]	3.5–8	1–3	0	0
Polydispersity [28]	4.2–7	2.5–3.5	1.5	2.5–3.5
Molecular weight	~15,000	~25,000	~5000	~15,000
Tg (^o^C) [30]	130	140–150	90–110	140
Solubility [30]	Water	Alkali	Many solvents	Alkali
Prices (USD/T) [28]	180–500	260–500	280–520	200–300

**Table 3:** Lignin-based electrospun scaffolds applied for delivery of various bioactive molecules.

Composition	Fabrication Method	Loaded Drug(s)	Application	Reference
PCL/chitin-lignin shell/core fiber	Coaxial electrospinning	methylene blue, penicillin/streptomycin	drug release, wound dressing	[132]
PCL/lignin	Initial blending	MTT	tissue engineering	[133]
LNPs@PVA/PVP	Initial blending	paclitaxel	local anticancer therapy	[134]
chitin-lignin	Initial blending	melatonin, vitamin C, and beta-glucan	cosmetic	[23]
PVA/lignin	Initial blending	AgNPs	membrane filtration, antimicrobial fabrics, and wound dressing	[130]
lignin/CA	Initial blending	copper complexes	antimicrobial nanofibrous mats for hygienic applications	[131]

## Data Availability

Not applicable.

## Abbreviations

AM: additive manufacturing; CAD: computer-aided design; SLA: stereolithography; DLP: digital light processing; DIW: direct ink writing; FDM: fused deposition modeling; LNPs: lignin nanoparticles; PLA: polylactic acid; PVP: polyvinylpyrrolidone; PCL: polycaprolactone; PHB: polyhydroxybutyrate; PBS: polybutylene succinate; HPU: hydrophilic polyether-based polyurethane; GAN: Gantrez S-97; PEG: poly(ethylene glycol); ECH: epichlorohydrin; [Emim][Ac]: 1-ethyl-3-methylimidazolium acetate; LA: lignin amine, PVA: poly(vinyl alcohol); NPs-P-PAA: composite hydrogels comprising silver-lignin core-shell nanoparticles (Ag-LNPs), pectin, and acrylic acid; BC: bacterial cellulose; DHP: dehydrogenative polymer of coniferyl alcohol (DHP); EDC: *N*-(3-Dimethylaminopropyl)-*N′*-ethylcarbodiimide hydrochloride. GGMMA: methacrylated-O-acetyl-galactoglucomannan; MTT: 3-(4,5-Dimethyl-2-thiazolyl)-2,5-diphenyl-2H -tetrazolium bromide.
